# External negative pressure improves lung aeration in near-term rabbit kittens at risk of developing respiratory distress

**DOI:** 10.3389/fped.2024.1526603

**Published:** 2025-01-15

**Authors:** C. Diedericks, K. J. Crossley, D. Jurkschat, M. J. Wallace, I. M. Davies, P. J. Riddington, A. B. te Pas, M. J. Kitchen, S. B. Hooper

**Affiliations:** ^1^ The Ritchie Centre, Hudson Institute of Medical Research, Clayton VIC, Australia; ^2^ Department of Obstetrics and Gynaecology, Monash University, Clayton VIC, Australia; ^3^ School of Physics and Astronomy, Monash University, Clayton VIC, Australia; ^4^ Division of Neonatology, Department of Paediatrics, Leiden University Medical Centre, Leiden, Netherlands

**Keywords:** negative pressure ventilation, swaddling, chest wall, lung liquid, respiratory distress, phase contrast x-ray

## Abstract

**Introduction:**

As airway liquid is cleared into lung interstitial tissue after birth, the chest wall must expand to accommodate this liquid and the incoming air. We examined the effect of applying external positive and negative pressures to the chest wall on lung aeration in near-term rabbit kittens at risk of developing respiratory distress.

**Methods:**

Rabbit kittens (30 days; term ∼31 days) were randomised into *Control* and *Elevated Liquid (EL)* groups. Lung liquid was drained in *Control* kittens to simulate expected volumes following vaginal delivery. *EL* kittens had lung liquid drained before 30 ml/kg was returned to simulate expected volumes after caesarean section. Kittens were delivered, placed in a water-filled plethysmograph and the external pressure was adjusted to −6 (negative), 0 (atmospheric), or +6 (positive) cmH_2_O. Kittens were ventilated with an 8 ml/kg tidal volume and PEEP of 0 cmH_2_O and lungs imaged using phase contrast x-ray imaging.

**Results:**

Compared to external atmospheric pressures, external negative pressures expanded the chest (by 2100 ± 43 vs. 1805 ± 59 mm^2^; *Control* kittens; *P* = 0.028), directed tidal ventilation into lower, larger lung regions and increased functional residual capacity (FRC) levels in both *Control* (26.7 ± 2.0 vs. 12.6 ± 2.2 ml/kg; *P* < 0.001) and *EL* (19.6 ± 1.6 vs. 10.0 ± 2.9 ml/kg; *P* < 0.01) kittens. External positive pressures reduced FRC levels in *Control* (6.3 ± 0.8 vs. 12.6 ± 2.2 ml/kg; *P* < 0.05), but not in *EL* kittens, and directed tidal ventilation into upper lung regions.

**Discussion:**

External negative pressures increased lung aeration and resulted in a more evenly distributed tidal ventilation immediately after birth in near-term rabbit kittens, whereas external positive pressures reduced lung aeration and compliance.

## Introduction

Neonatal respiratory distress (RD) is commonly associated with preterm infants who are born with immature and surfactant-deficient lungs (1). However, RD also occurs in near-term and term infants and is the leading reason for admission into neonatal intensive care, accounting for 51.2% of infants admitted (2). However, the underlying cause of RD is very different in term and preterm infants, with the primary cause of RD in term infants being higher-than-expected volumes of liquid in the airways of those delivered by caesarean section without labour (3–6).

All liquid present in the airways at birth must be cleared into the surrounding pulmonary interstitial tissue, irrespective of whether sodium reabsorption or inspiratory efforts are responsible (7, 8). Airway liquid clearance results in pulmonary oedema that can last for hours after birth while the liquid is slowly cleared from the pulmonary interstitium via lymphatics and blood vessels (3, 8–10). As the presence of larger volumes of liquid in the airways at birth increases the degree of pulmonary oedema after birth, the adverse effects on lung function in the immediate newborn period are also increased (3).

To accommodate both the incoming air, which establishes the newly formed functional residual capacity (FRC), and the liquid that has moved from the airways into lung tissue, the chest wall must expand at birth (7). As the neonatal chest wall is highly compliant, it can easily expand to accommodate the newly formed FRC, but its capacity to expand is limited (7). Thus, if the volume of liquid in the airways at birth is greater than expected, such as in infants delivered by caesarean section without labour, the chest wall must expand further to accommodate the same FRC. However, there must be a point at which further chest wall expansion requires an intra-thoracic pressure that exacerbates the effects of pulmonary oedema and compromises lung function.

While chest wall expansion is an integral component of the respiratory transition at birth, its role has been largely overlooked as a factor contributing to respiratory function after birth. Some common delivery room practices such as tight swaddling can apply an external positive pressure of 5–10 cmH_2_O to the chest wall (11). As the application of external positive pressures (∼7 cmH_2_O) limits chest wall expansion and impairs gas exchange in near-term lambs, it is possible that over-swaddling can adversely affect respiratory function in newborn infants (12). Indeed, swaddling is associated with increased respiratory rates in infants, likely a compensatory mechanism resulting from a decreased FRC (11). Conversely, the application of external negative pressures can improve oxygenation in infants and can facilitate chest wall expansion, reduce mean airway pressure, increase oxygen delivery, and reduce the fraction of inspired oxygen (FiO_2_) requirement in near-term newborn lambs (12–14).

The mechanisms by which external thoracic pressures impact gas exchange after birth are unknown, but they most likely affect lung aeration and function. In this study, we have used phase-contrast x-ray imaging to investigate the effects of external positive and negative pressures on lung aeration in near-term rabbit kittens at risk of developing respiratory distress after birth. We hypothesised that external positive pressures would compress the chest wall, which would subsequently compress the lungs and reduce FRC levels, thereby compounding the adverse effects of elevated airway liquid volumes. We also hypothesised that external negative pressures would expand the chest wall and increase FRC levels, thereby reducing the adverse effects of elevated airway liquid.

## Materials and methods

### Ethical approval

All animal procedures were approved by the SPring-8 Animal Care and Monash Medical Centre Animal Ethics Committee and conducted in accordance with the National Health and Medical Research Council (NHMRC) code of practice for the care and use of animals for scientific purposes (15). Methodological reporting is provided per the ARRIVE guidelines (16).

### Experimental procedure

Pregnant Japanese White rabbit does at 30 days of gestational age (GA; term ∼31 days) were initially sedated with propofol (8 mg/kg bolus i.v., followed by 20 ml/kg/h Propofol, Nichi-Iko Pharmaceuticals Co. Ltd, Toyama, Japan). Anaesthesia was maintained following intubation with inhaled isoflurane (Isoflurane, 1.5%–4%, Viatrus Pharmaceuticals Inc., Tokyo, Japan) as previously described (3). Rabbit kittens were exteriorised via caesarean section, with the umbilical cord kept intact. The kittens were anesthetised with sodium secobarbital (250 µl i.p., Ional Sodium, Nichi-Iko Pharmaceuticals Co. Ltd, Toyama, Japan) and intubated via a tracheostomy (18G intracath; BD Australia). After intubation, airway liquid was drained from the endotracheal tube using a 1 ml syringe. Kittens were randomised into two treatment groups, *Control* or *Elevated Liquid (EL)*. *Control* kittens received no further treatment, simulating expected airway liquid volumes following vaginal delivery with labour. Following airway liquid draining, *EL* kittens had 30 ml/kg Hartmann's solution added back via the endotracheal tube to simulate expected airway liquid volumes following elective caesarean section (liquid volumes drained and added are shown in Table 1). It is expected that ∼7 ml/kg remains in the distal airways following drainage, therefore, we expect that *Control* kittens had ∼7 ml/kg in their airways, and *EL* kittens had ∼37 ml/kg liquid in their airways (4, 17). The endotracheal tube was blocked to prevent loss of airway liquid before the onset of ventilation and imaging.

**Table 1:** Subject characteristics in *Control* and *Elevated Liquid* (*EL)* kittens**.**

Groups	Control			Elevated liquid			Sig.
	Positive	Atmospheric	Negative	Positive	Atmospheric	Negative	
Weight (g)	35 ± 5	35 ± 6	36 ± 7	40 ± 10	35 ± 8	38 ± 8	ns
Lung liquid drained (ml)	0.14 ± 0.04	0.15 ± 0.08	0.24 ± 0.10	0.38 ± 0.66	0.12 ± 0.07	0.18 ± 0.02	ns
Lung liquid added (ml)	N/A			1.1 ± 0.2	1.0 ± 0.2	1.0 ± 0.2	ns

The data were analysed with a one-way ANOVA. Mean ± SD.

### Ventilation protocol

**Figure 1:**
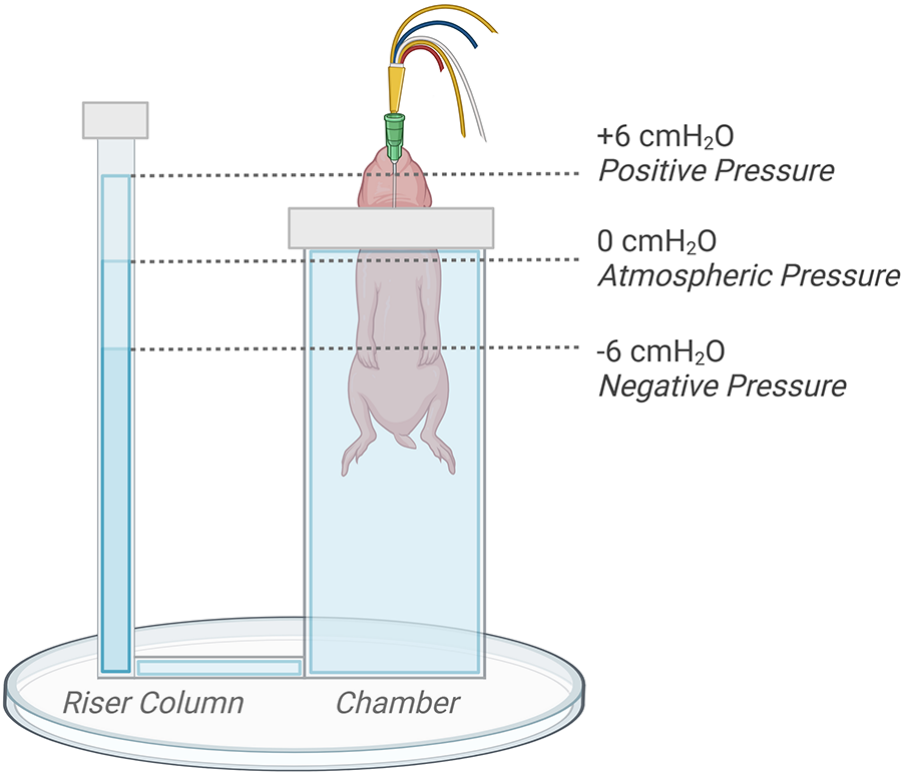
Kitten positioning and plethysmograph experimental set-up. The water level in the riser tube of the plethysmograph was adjusted by +6 (positive), −6 (negative), or 0 (atmospheric) cm relative to the middle of the chest to regulate externally applied pressures. *Figure created on BioRender.com*.

Following delivery, kittens were placed upright and head-out in the main chamber of a U-shaped, water-filled (39 ℃) plethysmograph located in the imaging hutch as previously described (Figure 1) (3, 7). The main chamber was sealed using a rubber diaphragm that fitted snuggly around the neck of the kitten, leaving only the riser tube open to the atmosphere. Kittens were further block randomised into experimental groups allocated to receive an external pressure of either +6 cmH_2_O (*Control n* = 8; *EL n* = 8), 0 cmH_2_O (*Control n* = 7; *EL n* = 7), or −6 cmH_2_O (*Control n* = 7; *EL n* = 7). These external pressures were achieved by adjusting the water level in the riser column of the plethysmograph; they were adjusted to be 6 cm above (+6 cmH_2_O), 6 cm below (−6 cmH_2_O), or at the level of the middle of each kittens' chest (0 cmH_2_O) to regulate the external pressure applied (Figure 1).

The endotracheal tube was connected to a custom-built ventilator (18) and, once the allocated external pressures were applied, the hutch was closed and imaging commenced. Ventilation commenced with a sustained inflation (SI), with the SI pressure increasing incrementally by 2 cmH_2_O/step starting from 24 cmH_2_O. Then, when air first began to enter the lung, the incremental pressure increase was paused and this SI pressure was maintained until 20 ml/kg of air had entered the lungs, at which point the SI ceased. Kittens were then mechanically ventilated with a positive end-expiratory pressure (PEEP) of 0 cmH_2_O, inspiratory time of 650 ms, expiratory time of 750 ms, and a tidal volume (Vt) of 8 ml/kg measured directly from the plethysmograph as previously described (3). After ∼6 min, a stepwise PEEP recruitment procedure was undertaken, with PEEP incrementally adjusted from 0 cmH_2_O to 2, 4, 6, 4, 2, and 0 cmH_2_O. Throughout the experimental period, airway pressure, lung gas volume (measured from the plethysmograph), external pressure, expired CO_2_, and heart rate were recorded on LabChart 8 (PowerLab, ADInstruments, NSW, Australia). At the end of the experimental period, all kittens were euthanised with an overdose of sodium secobarbital (3 mg i.p. Ional Sodium, Nichi-Iko Pharmaceuticals Co. Ltd, Toyama, Japan).

### Phase-contrast x-ray imaging

The study was conducted in experimental hutch 3 of beamline 20B2 in the Biomedical Imaging Centre of the SPring-8 Synchrotron Facility in Japan. The entire thoracic cavity and upper abdomen of each rabbit kitten was imaged using phase-contrast and a synchrotron x-ray radiation source tuned to an energy of 24 keV, as previously described (3, 4). The x-ray source-to-sample distance was ∼210 m, and the sample-to-detector distance was ∼2 m. A detector (Hamamatsu ORCA Quest qCMOS C15550-20UP) was coupled to a 25-μm thick gadolinium oxysulfide (Gd2O2S:Tb+) powdered phosphor and a tandem lens system that provided an effective pixel size of 17.86 μm and an active field of view of 39 (W) × 29 (H) mm^2^*.* Image acquisition was triggered by the ventilator at the onset of each inflation, which triggered a train of 7 images at 20 Hz ensuring that image acquisition was synchronised with each ventilation cycle. Flat-field and dark-current images were acquired after each kitten was imaged to allow for the correction of variations in the beam intensity and detector dark current signal.

### Phase-contrast x-ray image analysis

#### Lung gas volumes

Regional lung gas volumes were measured by phase-contrast x-ray imaging as previously described (3, 4, 19). Kittens were imaged while upright in the plethysmograph and images of the chest were divided into quadrants: upper left (UL), upper right (UR), lower left (LL), and lower right (LR). The 7th rib was used as the boundary to differentiate the upper and lower quadrants. The regional images were used to measure lung gas volumes at peak FRC, peak inflation, and tidal volume (Vt). The uniformity of lung aeration was assessed by calculating the 20-s trailing average of the coefficient of variation at 5 min after the SI, with lower values indicating more uniform aeration (20).

#### Tidal volume

The tidal volume heatmaps were calculated using a regional adaption of the method previously presented (19). A 256-pixel wide scanning window was used to calculate the regional projected thickness of air throughout the lungs at both FRC and peak inspiration. The pixel-wise sum of each regional projected thickness image was normalised to the total lung air volume calculated using a region that covered the whole chest. Regional tidal volume was then obtained by subtracting the resulting FRC image from the resulting peak inspiration image. This process was repeated for all kittens and coloured on a scale bounded by the minimum and maximum regional tidal volume.

#### Chest wall measurements

Image J (National Institute of Health, Maryland, USA) (21) was used to measure the thoracic area before aeration and at both maximum FRC and inspiration using a manually drawn region of interest (ROI) bounded by the 1st and 9th rib and the “measure” function. Between the left and right 9th rib, the ROI was drawn to the diaphragm peak in the FRC images, with the vertebra that matched the diaphragm height used in the unaerated images. The thoracic area was corrected for the length between the 1st and 5th vertebrae measured for each kitten on ImageJ to adjust for body size. The radius of diaphragm curvature at FRC was calculated by fitting a circular ROI to the outline of the diaphragm and corrected for the length between the 1st and 5th vertebrae. A single observer (CD) was blinded while analysing phase contrast x-ray images on ImageJ.

### Physiological data analysis

Physiological parameters measured on LabChart v8 (ADInstruments, Sydney, Australia) were analysed (20-s epochs every 1 min for 6 min; starting immediately following the sustained inflation). Dynamic respiratory system compliance (*C*_RS_) was calculated from ventilation parameters [*C*_RS_ = Vt (PIP − PEEP)]. As expired CO_2_ (relative measure) was measured using a CO_2_ analyser located in the ventilator, not at the mouth opening, expired CO_2_ data was time-shifted to synchronise the expired CO_2_ recording with other physiological data as previously described (9).

### Statistical analysis

A power analysis was conducted to determine the required sample size (∼7 kittens per group) for the primary outcome of thoracic expansion based on a previous study (3). The power was set at 0.8, and the type one error rate to 0.5. This study aimed to investigate the impact of positive (+6 cmH_2_O) and negative (−6 cmH_2_O) external pressures on lung aeration in *Control* and *EL* kittens compared to controls at atmospheric pressure (0 cmH_2_O). All data were assessed for normality on IBM SPSS Statistics (Version 29) and logarithmically transformed if required. Continuous data were analysed using a repeated measures general linear model followed by a Sidak-corrected estimated means pairwise comparisons in IBM SPSS Statistics. Comparisons were made to analyse the effect of external pressure in *Control* and *EL* kittens, as well as the impact of airway liquid volumes on lung aeration. The thoracic area before lung aeration and at maximum FRC were analysed using a one-way ANOVA. Regional tidal ventilation data were analysed with a repeated-measures two-way ANOVA. Subject characteristics (Table 1) and plethysmograph pressure were presented as mean ± SD and all remaining data were presented as mean ± SEM. *P* ≤ 0.05 were considered statistically significant.

## Results

### Animal inclusion and subject characteristics

Nine rabbit does and 57 kittens were initially used for this study, but one doe and her litter (9 kittens) were excluded due to premature delivery. Four kittens from the remaining 8 does were excluded due to surgical/imaging complications. All remaining kittens (44 kittens from 8 does) were included in the analysis, and all had similar body weights, volume of lung liquid drained, and volume of lung liquid added to the airways of *EL* kittens, between groups (Table 1).

### Plethysmograph pressure

During the application of external atmospheric pressure, the mean (SD) pressure in the plethysmograph was 0.5 ± 1.0 cmH_2_O (*Control*) and 0.7 ± 0.7 cmH_2_O (*EL*) at the end of the experimental period. During application of external positive pressures, the pressure in the plethysmograph was 5.5 ± 0.6 cmH_2_O in *Control* kittens and 5.7 ± 0.8 cmH_2_O in *EL* kittens. Before lung aeration, the external negative pressures applied were −5.5 ± 0.1 cmH_2_O below atmospheric pressure in *Control* kittens and −5.7 ± 0.2 cmH_2_O in *EL* kittens. However, 5 min after the sustained inflation (SI), the pressure in the plethysmograph had increased to −2.7 ± 0.8 cmH_2_O in *Control* kittens and −3.7 ± 0.3 cmH_2_O in *EL* kittens. Both external positive and negative pressures were significantly different from the atmospheric pressure group (*P* < 0.0001).

### Air distribution during tidal ventilation

**Figure 2:**
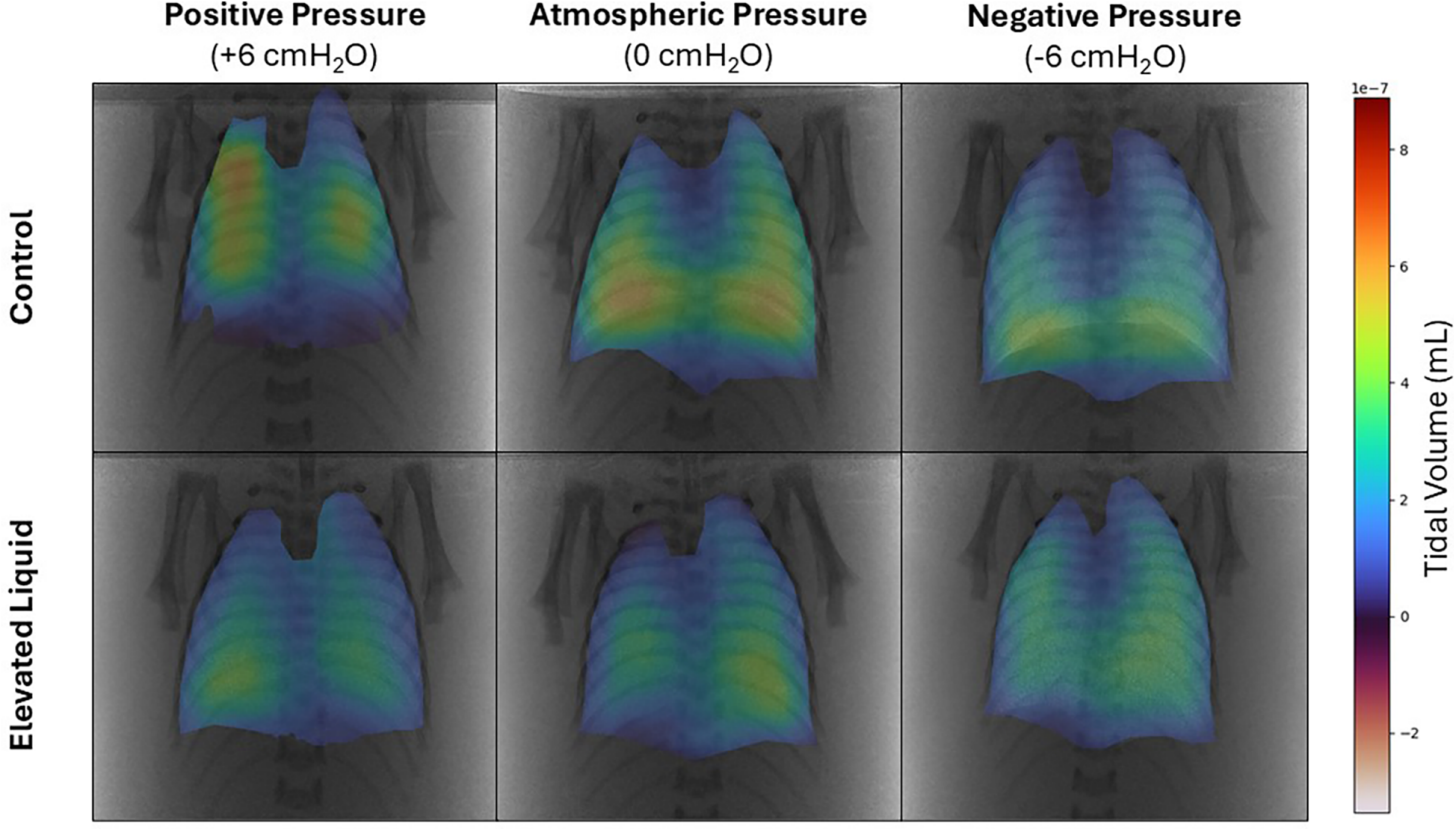
Representative colour maps showing the regional distribution of air during tidal ventilation. Regional tidal volume was calculated by subtracting functional residual capacity from peak gas volume at the end of the experimental period in *Control* and *Elevated Liquid (EL)* kittens exposed to external positive, atmospheric, and negative pressures.

**Figure 3:**
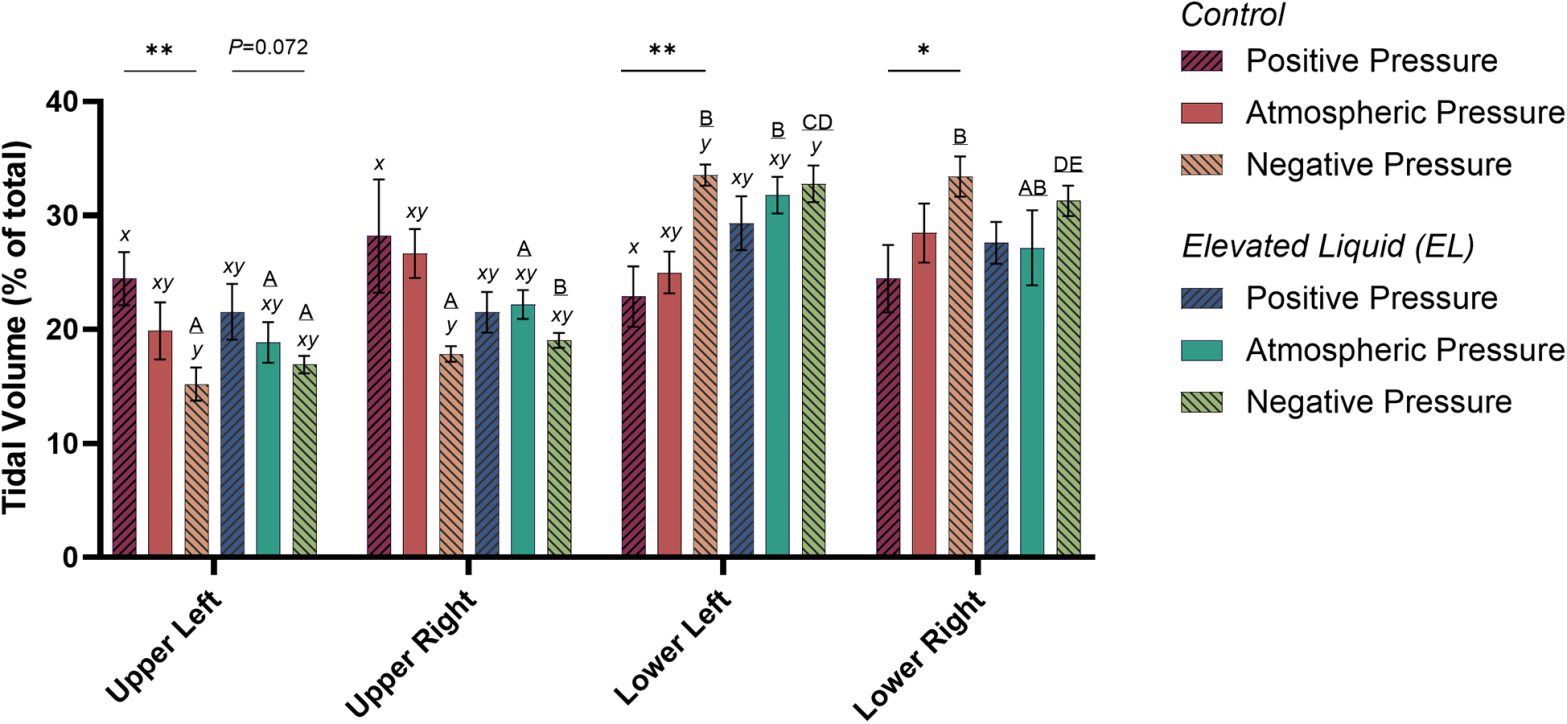
Regional distribution of tidal volume in the upper left, upper right, lower left, and lower right quadrants of the lung during tidal ventilation (% of total). Bars that do not share a common letter are significantly different from each other (*P* ≤ 0.05); A, B, C, D & E, differences between lung regions within treatment groups (repeated measures one-way ANOVA with Tukey's multiple comparisons); *x*, *y*, differences between treatment groups within lung regions (one-way ANOVA with Tukey's multiple comparisons). Asterisks indicate linear regression significance from zero evaluating the relationship between external pressure and distribution of tidal ventilation. Mean ± SEM.

In *Control* kittens, the distribution of tidal ventilation within the lung was significantly related to the external pressure applied. As the level of external pressure decreased, the distribution of ventilation was redirected towards the lower lung regions (LL, *R*^2^ = 0.30; *P* = 0.008; and LR, *R*^2^ = 0.23; *P* = 0.02; Figures 2, 3) and away from the upper lung regions (UL, *R*^2^ = 0.32; *P* = 0.006; and UR, *R*^2^ = 0.15; *P* *=* 0.072). While this relationship was not significant in *EL* kittens, the trend appeared to be similar for the left lung, but not for the right lung (Figures 2, 3).

#### External pressures in *Control* kittens

In *Control* kittens, the proportion of tidal volume entering the UL and UR lung regions were significantly greater in kittens exposed to positive compared to negative external pressures (UL; 24.5 ± 2.3% vs. 15.2 ± 1.4%; *P* = 0.047; UR; 28.2 ± 5.0% vs. 17.9 ± 0.7%; *P* = 0.017). In contrast, the percentage of tidal volume entering the LL lung region was significantly greater in kittens exposed to negative compared to positive external pressures (33.5 ± 0.9% vs. 22.9 ± 2.6%; *P* = 0.013). Similarly, the percentage of tidal volume entering the LR lung region tended to be greater in kittens exposed to negative compared to positive external pressures, although this difference was not quite significant (33.4 ± 1.8 vs. 24.5 ± 2.9%; *P* = 0.060; Figure 3). The percentage of tidal volume entering the lung quadrants in kittens exposed to atmospheric pressure were not significantly different compared to kittens exposed to negative and positive external pressures (Figures 2, 3).

#### External pressures in *EL* kittens

In *EL* kittens, the proportion of tidal volume entering the lung quadrants were not significantly different between kittens exposed to positive, atmospheric or negative external pressures (Figures 2, 3).

When *EL* kittens were exposed to external atmospheric pressures, a significantly greater proportion of tidal volume entered the LL compared to the UL or UR regions of the lungs (LL, 31.8 ± 3.3%; UL, 18.9 ± 1.8%; UR, 22.2 ± 1.3%; *P* < 0.05; Figure 3). Similarly, external negative pressure increased the proportion of tidal ventilation in the lower compared to the upper lung regions (LL, 32.8 ± 1.6; LR, 31.3 ± 1.3; UL, 16.9 ± 0.8; UR, 19.0 ± 0.6%; *P* < 0.01; Figure 3).

### Chest wall mechanics

#### Effect of elevated airway liquid volumes

**Figure 4:**
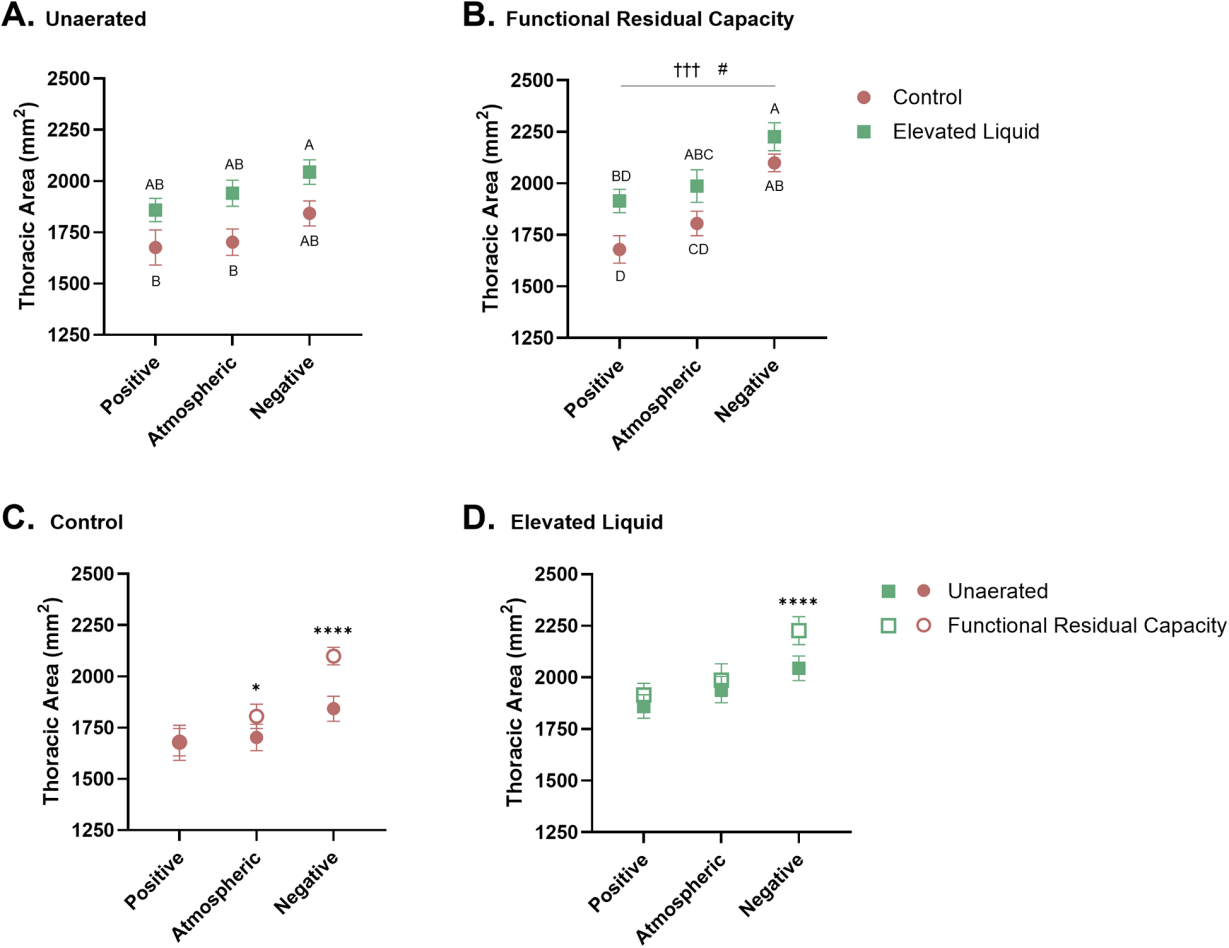
Thoracic area standardised by the length (mm^2^) between the 1–5th vertebrae. Thoracic area of *Control* and *Elevated Liquid (EL)* kittens are shown **(A)** before lung aeration and **(B)** at functional residual capacity (FRC); data were analysed with a one-way ANOVA followed by Tukey's multiple comparisons. Points that do not share a common letter are significantly different from each other (*P* ≤ 0.05). **(B)** presents a linear regression assessing the relationship between external pressure and thoracic area († indicates significance within the *Control* group, †††*P* < 0.001; and # indicates significant differences within the *EL* group, #*P* < 0.05). Thoracic area data before lung aeration and at FRC are shown in **(C)** *Control* and **(D)** *EL* kittens and were analysed with a repeated measures two-way ANOVA followed by Šídák's multiple comparisons. * indicates significance between unaerated and FRC thoracic areas in each treatment group. *P < 0.05, ****P < 0.0001. Mean ± SEM.

Thoracic area before lung aeration was significantly greater in *EL* kittens exposed to external negative pressures compared to *Control* kittens exposed to atmospheric (2,044 ± 60 vs. 1,703 ± 64 mm^2^; *P* = 0.013) and positive external pressures (2,044 ± 60 vs. 1,676 ± 85 mm^2^; *P* = 0.004; Figure 4A), but was not significantly different from thoracic area in *Control* kittens exposed to external negative pressures (2,044 ± 60 vs. 1,843 ± 61 mm^2^). Similarly, after lung aeration, the thoracic area (measured at FRC) was greater in *EL* kittens exposed to external negative pressures than *Control* kittens exposed to atmospheric (2,227 ± 67 vs. 1,805 ± 59 mm^2^; *P* = 0.001) and positive external pressures (2,227 ± 67 vs. 1,680 ± 67 mm^2^; *P* < 0.0001). There was no significant difference between thoracic area (measured at FRC) in *Control* and *EL* kittens exposed to external negative pressures. The thoracic area was also greater in *EL* kittens exposed to external atmospheric pressures than *Control* kittens exposed to external positive pressures, measured after lung aeration at FRC (1,987 ± 79 vs. 1,680 ± 67 mm^2^; *P* = 0.015; Figure 4B).

#### Effect of external pressures in *Control* kittens

Before lung aeration, the thoracic area was similar in *Control* kittens exposed to external positive, atmospheric, and negative pressure, although tended to increase with decreasing pressure (Figure 4A). However, after lung aeration, thoracic area (measured at FRC) was significantly greater in *Control* kittens exposed to external negative pressures than kittens exposed to positive (2,100 ± 43 vs. 1,680 ± 67 mm^2^; *P* = 0.0004) and atmospheric (2,100 ± 43 vs. 1,805 ± 59 mm^2^; *P* = 0.028; Figure 4B) external pressures.

In *Control* kittens, aeration of the lung increased the thoracic area (measured at FRC) in kittens exposed to both atmospheric (from 1,703 ± 64 to 1,805 ± 59 mm^2^; *P* = 0.013) and negative (from 1,843 ± 61 to 2,100 ± 43 mm^2^; *P* < 0.0001; Figure 4C) external pressures. However, thoracic area was similar before and after lung aeration in *Control* kittens exposed to external positive pressures. External pressures did not affect the curvature of the diaphragm or chest wall movement during tidal ventilation in *Control* kittens (Table 2).

**Table 2:** Measurements of ventilation, lung, and thoracic parameters.

Groups	Control			Elevated liquid			Sig.
	Positive	Atmospheric	Negative	Positive	Atmospheric	Negative	
Radius of diaphragm curvature (mm)	1.49 ± 0.07*a*	1.49 ± 0.06*a*	1.87 ± 0.10*ab*	1.58 ± 0.10*a*	1.66 ± 0.11*a*	2.28 ± 0.22*b*	*P* ≤ 0.05
Relative change in thoracic area during tidal ventilation (%)	1.19 ± 0.97	4.13 ± 0.74	2.48 ± 0.75	1.57 ± 0.68	1.36 ± 0.81	0.99 ± 1.07	ns
Peak inspiratory pressure (cmH_2_O)	31.9 ± 1.0*a*	12.4 ± 2.0*bc*	7.5 ± 1.0*c*	32.9 ± 0.7*a*	28.8 ± 1.2*a*	14.6 ± 1.7*b*	*P* ≤ 0.05
Uniformity of aeration (coefficient of variation)	0.47 ± 0.11	0.25 ± 0.04	0.20 ± 0.03	0.51 ± 0.24	0.43 ± 0.13	0.29 ± 0.04	ns
Lung bulging at FRC (Absent: Mild: Moderate)	7:0:1	6:1:0	6:1:0	8:0:0	6:1:0	7:0:0	ns
Lung bulging at Peak Gas Volume (Absent: Mild: Moderate)	7:0:1	6:1:0	5:2:0	8:0:0	6:0:1	7:0:0	ns

The radius of diaphragm curvature at functional residual capacity (FRC; mm), the coefficient of variation for uniformity of aeration at FRC 5 min after the sustained inflation (SI), peak inflation pressure at 5 min after SI (cmH_2_O) and change in thoracic area during tidal ventilation were analysed with a one-way ANOVA followed by Tukey's multiple comparisons. Lung bulging at FRC and peak gas volumes were analysed with Fisher's exact test. Significance is indicated by letters; if groups share a letter, they are not considered significantly different, and if they do not share a letter, they are significantly different. *P* ≤ 0.05 is considered significant. Mean ± SEM.

#### Effect of external pressures in *EL* kittens

Before lung aeration, the thoracic area of *EL* kittens was similar in kittens exposed to external positive, atmospheric, and negative pressures (Figure 4A), but tended to increase with decreasing pressure. However, after lung aeration, the thoracic area (measured at FRC) was significantly greater in kittens exposed to external negative pressures than external positive pressures (2,227 ± 67 vs. 1,915 ± 57 mm^2^; *P* = 0.013; Figure 4B).

Lung aeration significantly increased the thoracic area (measured at FRC) in *EL* kittens exposed to external negative pressures (2,044 ± 60 to 2,227 ± 67 mm^2^; *P* < 0.0001), but not in kittens exposed to external positive or atmospheric pressures (Figure 4D). External negative pressures increased the flattening of the diaphragm compared to external atmospheric (2.28 ± 0.22 vs. 1.66 ± 0.11 mm^2^; *P* = 0.011) and positive pressures (2.28 ± 0.22 vs. 1.58 ± 0.10 mm^2^; *P* = 0.002; Figure 4D), indicated by a larger radius of curvature (Table 2). External pressures did not affect chest wall movement during tidal ventilation in *EL* kittens (Table 2).

### Respiratory system compliance (*C*_RS_) and peak inflation pressures (PIP)

#### Effect of external pressures in *Control* kittens

**Figure 5:**
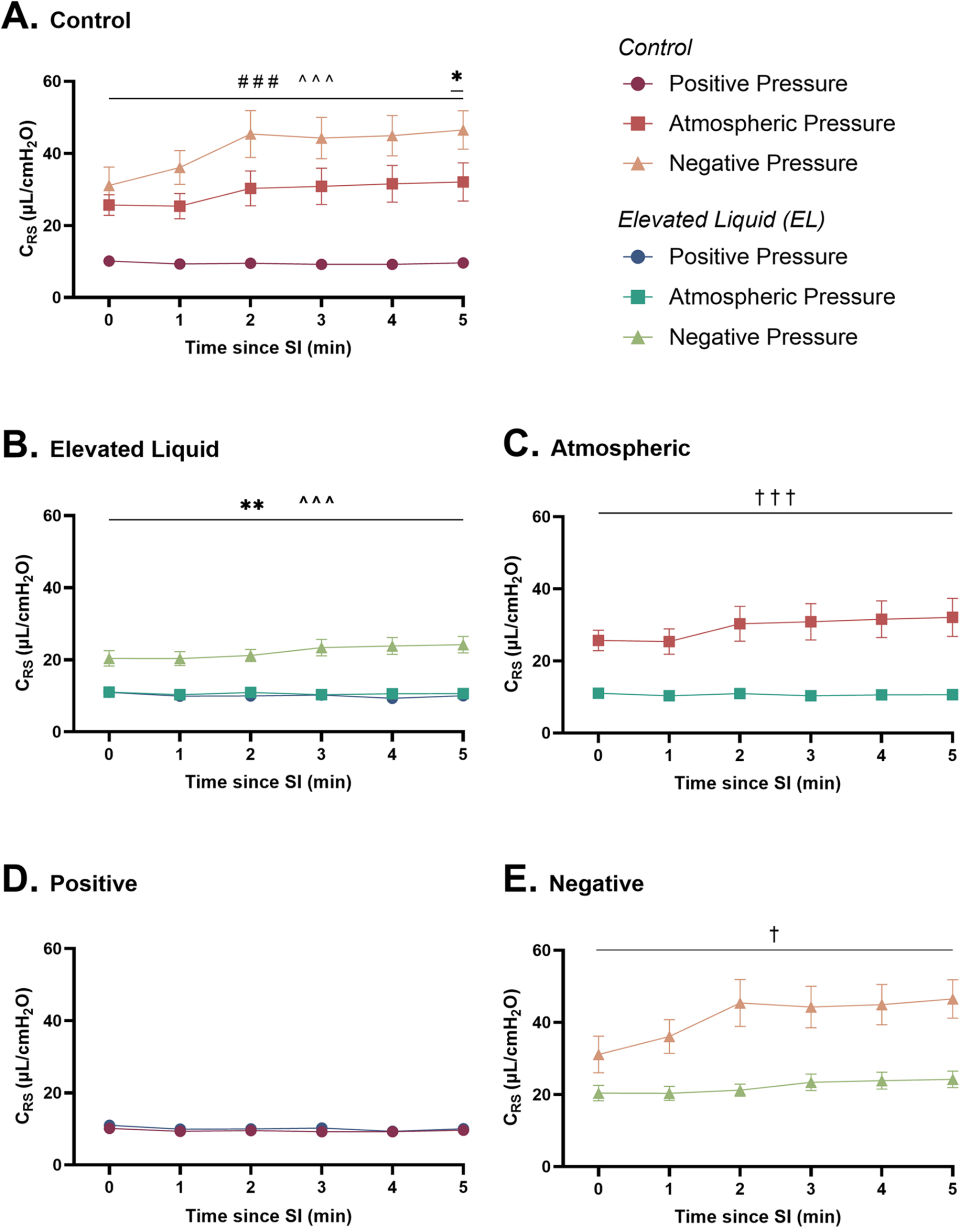
Respiratory system compliance (µl/cmH_2_O; *C*_RS_) after completion of the sustained inflation (SI). *C*_RS_ is shown in **(A)** *Control* kittens exposed to external positive (*n* = 8), atmospheric (*n* = 7), and negative (*n* = 7) pressures, as well as in **(B)** *Elevated Liquid* (*EL*) kittens exposed to external positive (*n* = 8), atmospheric (*n* = 7), and negative (*n* = 7) pressures. *C*_RS_ is compared in *Control* and *EL* kittens exposed to **(C)** atmospheric, **(D)** positive, and **(E)** negative external pressures. Data were logarithmically transformed, analysed with a general linear model with Sidak-corrected comparisons, and presented as mean ± SEM. A # indicates statistical significance between kittens in atmospheric and positive pressure (###*P* *<* 0.001), * indicates significant differences between kittens in atmospheric and negative pressure (*P ≤ 0.05, **P *<* 0.01), and ^ indicates significant differences between kittens in positive and negative pressures (^^^*P* *<* 0.001). Significance between *Control* and *EL* liquid kittens are indicated by daggers (†) in **(C)** and **(E)** (^†^*P* ≤ 0.05, ^†††^*P* *<* 0.001).

In *Control* kittens, external positive pressures reduced *C*_RS_ compared to external atmospheric pressures following the SI (9.6 ± 0.5 vs. 32.1 ± 5.3 µl/cmH_2_O at 5 min; *P* < 0.001; Figure 5A). In contrast, external negative pressures increased *C*_RS_ compared to atmospheric pressures at 5 min after the SI (46.5 ± 5.3 vs. 32.1 ± 5.3 µl/cmH_2_O; *P* = 0.02) and increased *C*_RS_ compared to positive pressures (46.5 ± 5.3 vs. 9.6 ± 0.5 µl/cmH_2_O at 5 min; *P* < 0.001; Figure 5A).

Furthermore, external positive pressures increased the PIP required to maintain Vt compared to both atmospheric (31.9 ± 1.0 vs. 12.4 ± 2.0 cmH_2_O; *P* < 0.0001) and negative external pressures (31.9 ± 1.0 vs. 7.5 ± 1.0 cmH_2_O; *P* < 0.0001; Table 2).

#### Effect of external pressures in *EL* kittens

In *EL* kittens, external negative pressures increased *C*_RS_ compared to both atmospheric (24.2 ± 2.3 vs. 10.6 ± 0.5 µl/cmH_2_O at 5 min; *P* < 0.001) and positive (24.2 ± 2.3 vs. 10.0 ± 0.6 µl/cmH_2_O at 5 min; *P* < 0.001; Figure 5B) external pressures. *C*_RS_ was similar in *EL* kittens exposed to positive and atmospheric external pressures.

PIP was significantly lower in *EL* kittens exposed to external negative pressures than positive (14.6 ± 1.7 vs. 32.9 ± 0.7; *P* < 0.0001) or atmospheric (14.6 ± 1.7 vs. 28.8 ± 1.2; *P* < 0.0001; Table 2) external pressures.

#### Effect of elevated airway liquid volumes

*C*_RS_ was increased after the SI in *Control* compared to *EL* kittens exposed to atmospheric (32.1 ± 5.3 vs. 10.6 ± 0.5 µl/cmH_2_O at 5 min; *P* < 0.001; Figure 5C) and negative (46.5 ± 5.3 vs. 24.2 ± 2.3 µl/cmH_2_O at 5 min; *P* < 0.001; Figure 5E) external pressures. However, *C*_RS_ was similar in *Control* and *EL* kittens exposed to external positive pressures (Figure 5D).

The required PIP levels were significantly higher in *EL* than in *Control* kittens exposed to atmospheric (28.8 ± 1.2 vs. 12.4 ± 2.0; *P* < 0.0001) and negative (14.6 ± 1.7 vs. 7.5 ± 1.0; *P* = 0.007) external pressures but were not different in kittens exposed to external positive pressures (Table 2).

### Lung aeration

#### Effect of external pressures in *Control* kittens

**Figure 6:**
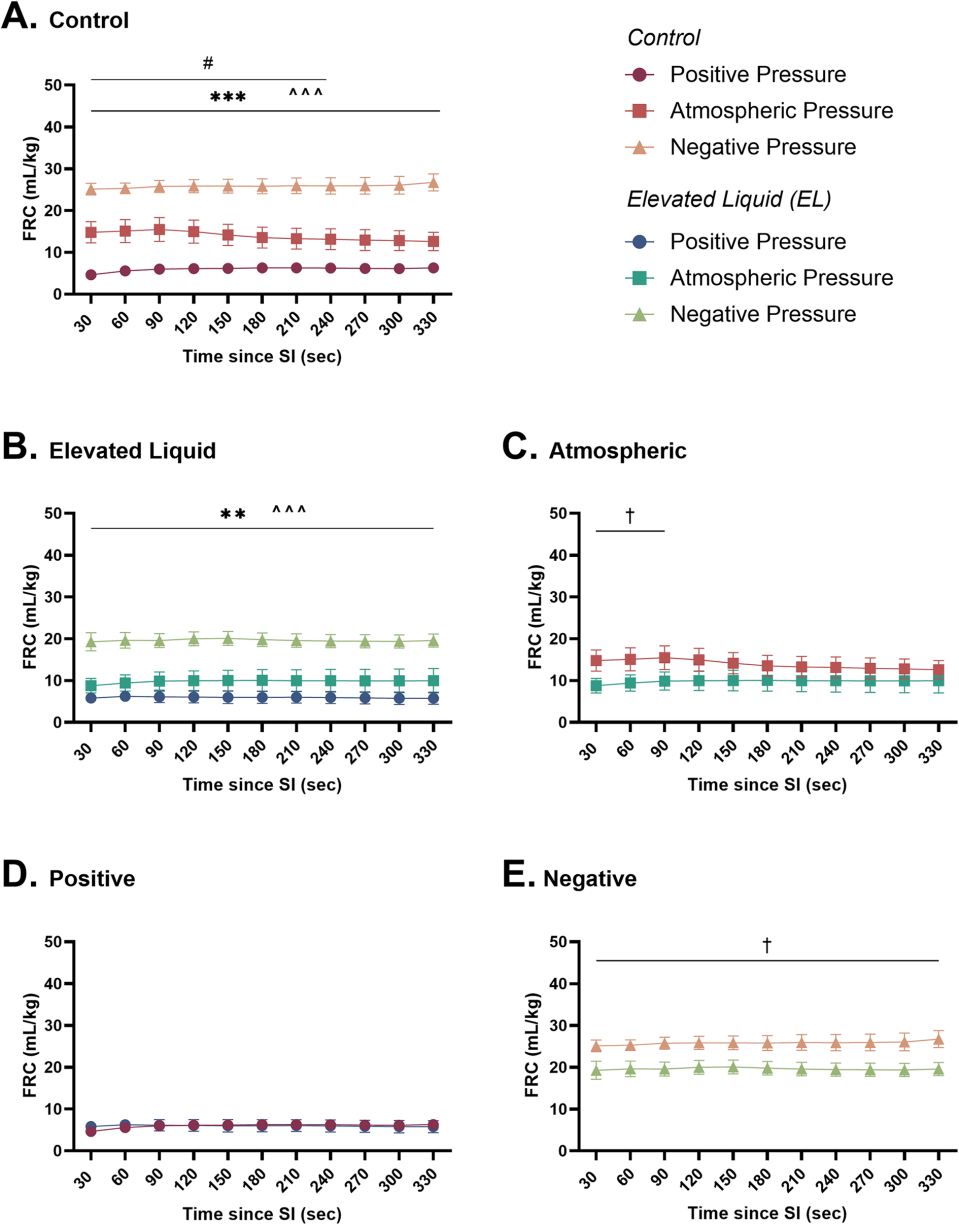
Functional residual capacity (ml/kg; FRC) after completion of the sustained inflation (SI). FRC is shown in **(A)** *Control* kittens exposed to external positive (*n* = 8), atmospheric (*n* = 6), and negative (*n* = 7) pressures, as well as in **(B)** *Elevated Liquid* (*EL*) kittens exposed to external positive (*n* = 8), atmospheric (*n* = 7), and negative (*n* = 7) pressures. FRC is compared in *Control* and *EL* kittens exposed to **(C)** atmospheric, **(D)** positive, and **(E)** negative external pressures. Data were analysed with a general linear model with Sidak-corrected comparisons and presented as mean ± SEM. A # indicates statistical significance between kittens in atmospheric and positive pressure (#*P* ≤ 0.05), * indicates significant differences between kittens in atmospheric and negative pressure (**P *<* 0.01, ***P *<* 0.001), and ^ indicates significant differences between kittens in positive and negative pressures (^^^*P* *<* 0.001). Significance between *Control* and *EL* liquid kittens are indicated by daggers (†) in **(C)** and **(E)** (^†^*P* *<* 0.05).

In *Control* kittens, external positive pressures reduced FRC levels compared to external atmospheric pressures after the SI (6.2 ± 0.9 vs. 13.1 ± 2.5 ml/kg at 240 s; *P* = 0.04; Figure 6A) but did not affect the presence of lung bulging or the uniformity of lung aeration (Table 2). Conversely, external negative pressures increased FRC levels compared to kittens exposed to external atmospheric pressures (26.7 ± 2.0 vs. 12.6 ± 2.2 ml/kg at 330 s; *P* < 0.001; Figure 6A). Despite increasing FRC levels, external negative pressures did not increase the presence of lung bulging at either FRC or peak inflation (Table 2). Neither external positive nor negative pressures influenced the uniformity of lung aeration compared to external atmospheric pressures at FRC in *Control* kittens (Table 2).

#### Effect of external pressures in *EL* kittens

In *EL* kittens, external negative pressures significantly increased FRC levels compared to *EL* kittens exposed to atmospheric pressure (19.6 ± 1.6 vs. 10.0 ± 2.9 ml/kg at 330 s; *P* = 0.003; Figure 6B). Increased FRC levels were not associated with an increased presence of lung bulging at either FRC or peak inflations and the uniformity of aeration was not influenced by external negative pressure (Table 2). In kittens exposed to external positive pressures, FRC levels, the uniformity of lung aeration at FRC, and the presence of lung bulging were not different compared to kittens exposed to external atmospheric pressures (Figure 6B; Table 2).

#### Effect of elevated airway liquid volumes

FRC levels were higher in *Control* than *EL* kittens exposed to external atmospheric pressures (from 30 to 90 s; 15.5 ± 2.9 vs. 10.0 ± 2.2 ml/kg at 90 s; *P* = 0.035; Figure 6C) and negative pressure (26.7 ± 2.0 vs. 19.6 ± 1.6 ml/kg at 330 s; *P* = 0.012; Figure 6E). When exposed to an external positive pressure, FRC levels were similar in *Control* and *EL* kittens (Figure 6D). The uniformity of lung aeration at FRC and presence of lung bulging was not different in *Control* and *EL* kittens exposed to either external atmospheric, positive, or negative pressures (Table 2).

### Expired CO_2_ levels

#### Effect of external pressures in *Control* kittens

**Figure 7:**
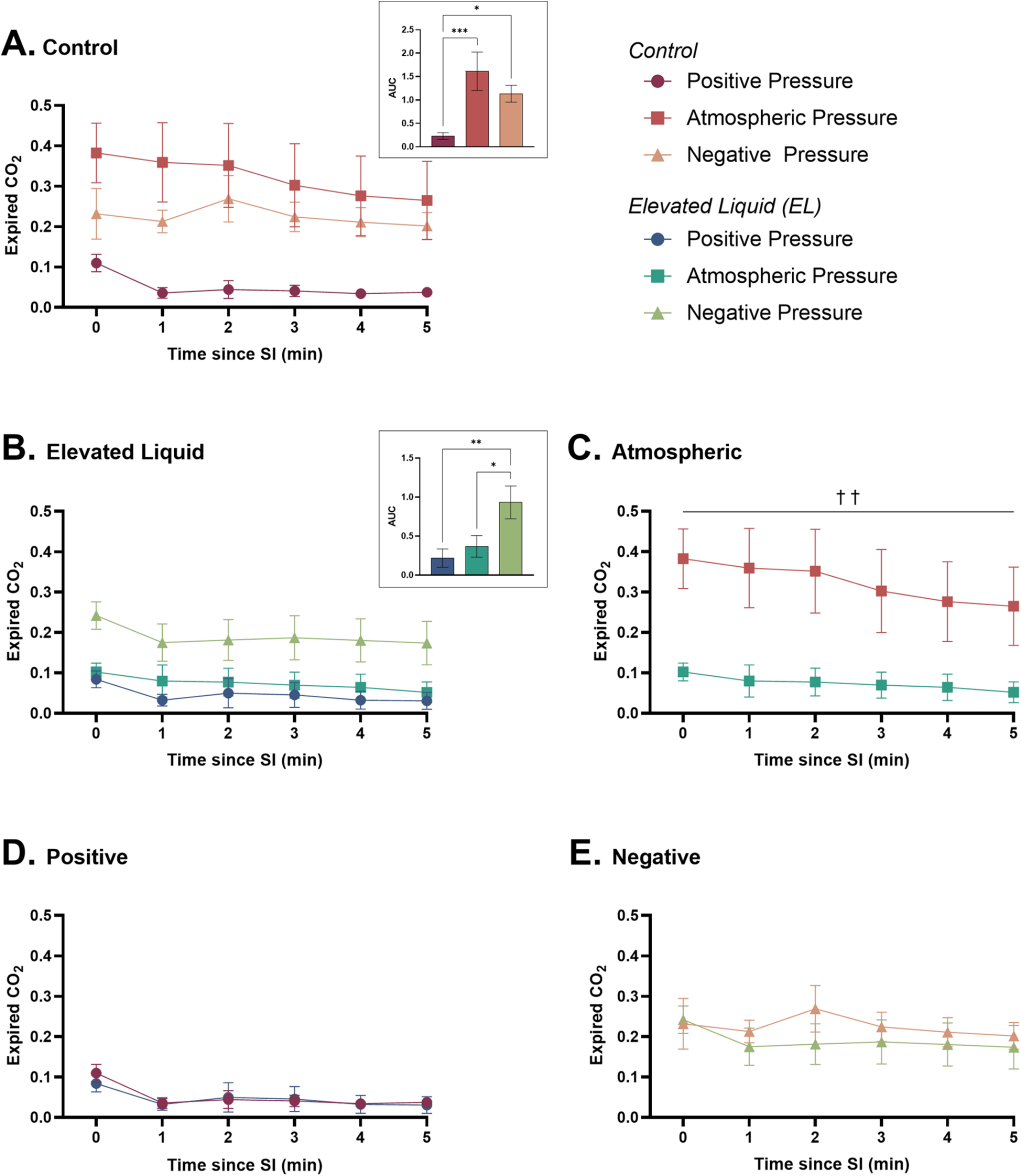
Expired CO_2_ (relative measure) after completion of the sustained inflation (SI). Expired CO_2_ is shown in **(A)** *Control* kittens exposed to external positive (*n* = 8), atmospheric (*n* = 7), and negative (*n* = 7) pressures, as well as in **(B)** *Elevated Liquid* (*EL*) kittens exposed to external positive (*n* = 8), atmospheric (*n* = 7), and negative (*n* = 7) pressures. Expired CO_2_ is compared in *Control* and *EL* kittens exposed to **(C)** atmospheric, **(D)** positive, and **(E)** negative external pressures. Area under the curve (AUC) data were analysed with a one-way ANOVA followed by Tukey's multiple comparisons (A & B) or an unpaired *t*-test (C, D, & E). Mean ± SEM. Significance is shown by AUC graphs (A & B) or daggers (†) in **(C)** (*P < 0.05, **P < 0.01, ***P < 0.001, ^††^*P* < 0.01).

In *Control* kittens, an external positive pressure reduced expired CO_2_ levels compared to kittens in both external atmospheric (AUC = 0.23 ± 0.07 vs. 1.61 ± 0.41; *P* = 0.0004) and negative pressures (AUC = 0.23 ± 0.07 vs. 1.13 ± 0.18; *P* = 0.029). Expired CO_2_ levels were not different between *Control* kittens exposed to external atmospheric and negative pressures (Figure 7A).

#### Effect of external pressures in *EL* kittens

Expired CO_2_ levels were significantly higher in *EL* kittens exposed to external negative pressures than external atmospheric (AUC = 0.93 ± 0.21 vs. 0.37 ± 0.14; *P* = 0.0004) and positive pressures (AUC = 0.93 ± 0.21 vs. 0.22 ± 0.12; *P* = 0.0004). There were no differences in expired CO_2_ levels between *EL* kittens exposed to external positive and atmospheric pressures (Figure 7B).

#### Effect of elevated airway liquid volumes

Expired CO_2_ levels were significantly higher in *Control* than *EL* kittens exposed to external atmospheric pressures (area under the curve = 1.63 ± 0.41 vs. 0.37 ± 0.14; *P* = 0.005; Figure 7C). Expired CO_2_ levels were not different in *Control* and *EL* kittens exposed to external positive (Figure 7D) and negative pressures (Figure 7E).

### Positive end-expiratory pressures vs. external negative pressures; effect on FRC

#### *Control* kittens

**Figure 8:**
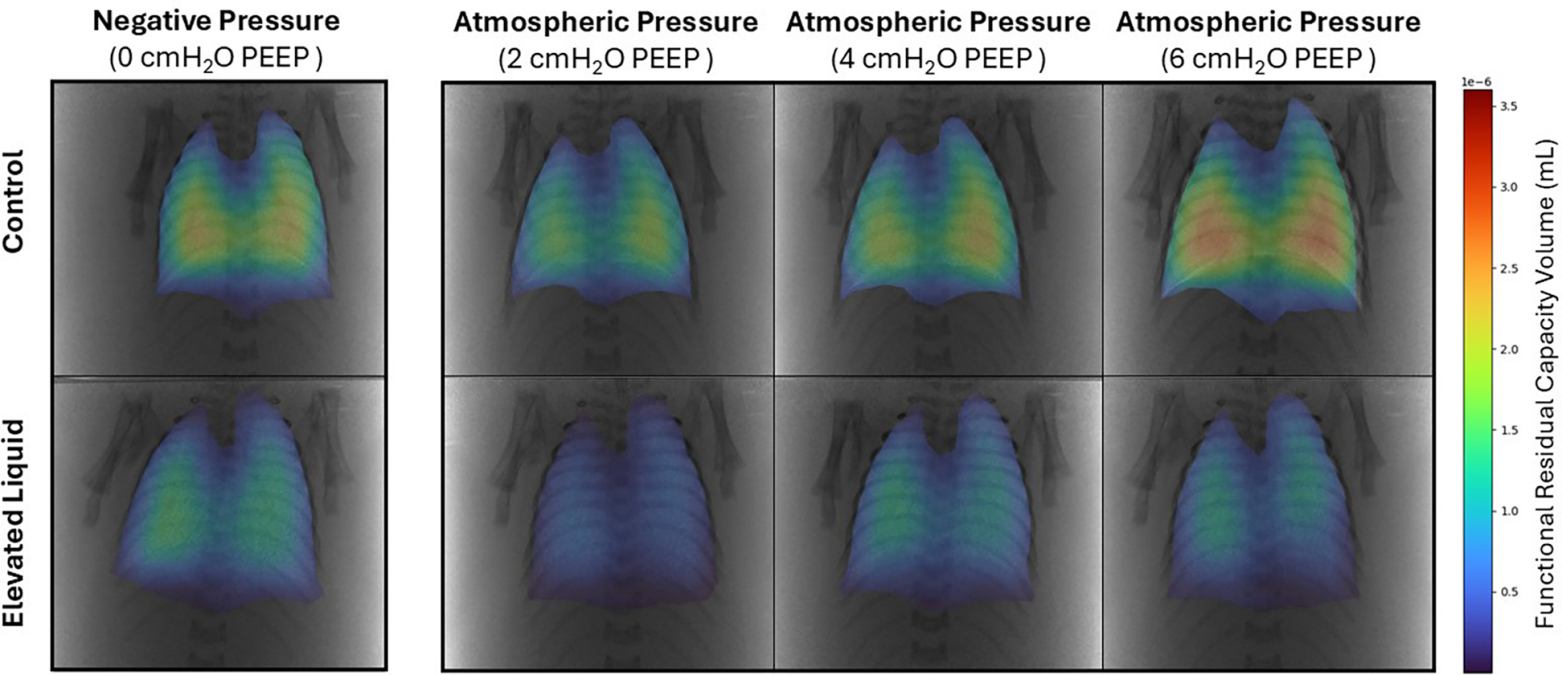
Representative colour maps of regional functional residual capacity (FRC; ml/kg) in *Control* and *Elevated Liquid (EL)* kittens. FRC is shown in kittens exposed to external negative pressures (−6 cmH_2_O) with 0 cmH_2_O positive end-expiratory pressure (PEEP), and external atmospheric pressures (0 cmH_2_O) with 2, 4, and 6 cmH_2_O PEEP.

**Figure 9:**
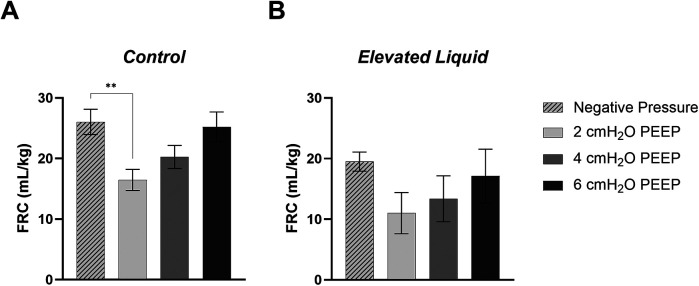
Functional Residual Capacity (FRC; ml/kg) levels in **(A)** *Control* and **(B)** *Elevated Liquid (EL)* kittens. FRC is shown in kittens exposed to external negative pressures (−6 cmH_2_O) with 0 cmH_2_O positive end-expiratory pressure (PEEP), and external atmospheric pressures (0 cmH_2_O) with 2, 4, and 6 cmH_2_O PEEP. 2, 4, and 6 cmH_2_O PEEP groups were compared to the external negative pressure group with a one-way ANOVA followed by Dunnett's multiple comparisons and presented as mean ± SEM. **P < 0.01.

In *Control* kittens, the FRC levels achieved when applying an external negative pressure (26 ± 2 ml/kg) with 0 PEEP were similar to that achieved when applying external atmospheric pressures with a PEEP of 4 (20 ± 2 ml/kg) and 6 cmH_2_O (25 ± 2 ml/kg) (Figures 8, 9A). While a PEEP of 4 cmH_2_O tended to result in lower FRCs than external negative pressures, this difference was not significant, whereas the FRC values achieved with a PEEP of 2 cmH_2_O were significantly lower than those achieved with negative pressure (26 ± 2 vs. 16 ± 2 ml/kg; *P* = 0.009; Figures 8, 9A).

#### *EL* kittens

In *EL* kittens, FRC levels were similar between kittens exposed to external negative pressures and kittens exposed to external atmospheric pressures with a PEEP of 2, 4, or 6 cmH_2_O (Figures 8, 9B). However, PEEP levels of 2 and 4 cmH_2_O in external atmospheric pressure tended to achieve lower FRC levels than external negative pressure.

## Discussion

The findings of this study add to the growing body of evidence indicating that applying external pressures to the chest wall can have a major impact on respiratory function in newborns. This study demonstrated that the application of external negative pressures expanded the chest wall, increased *C*_RS_ and FRC levels, and redirected tidal ventilation to the larger lower lobes of *Control* and *EL* kittens. Thus, external negative pressures can improve lung aeration and the distribution of ventilation in the immediate newborn period in near-term rabbit kittens, particularly in kittens with elevated airway liquid. In contrast, external positive pressures significantly reduced *C*_RS_ and lung aeration (FRC levels) and redirected tidal ventilation into the upper lobes of *Control* kittens. As swaddling can apply external pressures of ∼5–10 cmH_2_O (11), which are similar to the pressures applied in this study, over-swaddling infants in the immediate newborn period should be avoided.

### Air distribution during tidal ventilation

The application of external pressures had a major impact on the regional distribution of air during tidal ventilation. External negative pressures directed the distribution of tidal ventilation into the larger lower lung quadrants in both *Control* and *EL* kittens, which may partly explain the increase in *C*_RS_ associated with negative external pressures. Combined with the lower PIPs required to achieve a set tidal volume, external negative pressures would be expected to reduce the risk of ventilation-induced lung injury. Furthermore, as the lower lobes are larger and have a greater surface area (22), directing tidal ventilation into the lower lobes likely explains the increased oxygenation that occurs with external negative pressures (12, 13, 23). In contrast, in *Control* kittens, external positive pressures redirected the distribution of tidal ventilation from the lower lung quadrants into the upper lung quadrants during mechanical ventilation, although this was less evident in *EL* kittens. As the upper lobes of the lung are considerably smaller than the lower lobes (22), this may partly explain the decrease in *C*_RS_ and FRC levels associated with external positive pressures. As we were using a set tidal volume (8 ml/kg), forcing air to preferentially enter the upper lobes increases the risk of over-expansion and injury in those lobes.

It is also possible that the effect of external pressures on the distribution of tidal ventilation within the lung was in part due to pressures applied to the abdomen. For instance, in addition to the effect on the chest wall, a positive external pressure would also be expected to pressurise the abdomen and displace the diaphragm higher into the chest, which may cause a differential decrease in FRC and compliance across the lung, with the decrease being greatest in the lower lobes and least in the upper lobes.

### Chest wall mechanics

As the neonatal chest wall is highly compliant (24), it can easily expand to accommodate both the volume of air that fills the airways and the liquid that has been cleared from the airways into the surrounding lung tissue during lung aeration (7, 8, 12). However, when infants are delivered with greater than expected volumes of liquid in their airways, which likely occurs in infants delivered by caesarean section without labour, the chest wall must expand to a greater degree to accommodate that liquid and the same volume of air (FRC). While the chest wall is highly compliant, its capacity to expand is limited and, at some point, the intra-thoracic pressure required to expand the chest further must increase. Increased intra-thoracic pressures will reduce the volume of air that is retained in the lung at FRC, as well as the volume of air that can enter and leave the lung during tidal ventilation. Thus, elevated airway liquid reduces FRC and *C*_RS_ and is now considered to be the primary factor causing respiratory distress in term/near-term infants (3, 6).

We hypothesised that external negative pressures would expand the chest wall and increase FRC levels and *C*_RS_, particularly in kittens with elevated airway liquid, whereas positive pressures would compress and restrict chest wall movement. Interestingly, although we were unable to detect a significant effect of external pressure on chest wall dimensions, we found a significant negative linear relationship between external pressure and chest wall dimensions in both *Control* and *EL* kittens. While the application of external positive pressures did not affect chest wall movement during tidal ventilation, this is likely because the kittens were mechanically ventilated. They needed a higher peak inflation pressure to achieve the same tidal volume, thereby resulting in a similar chest wall movement. Thus, if we had used the same peak inflation pressures between groups, it is likely that we would have found that positive pressure reduces chest wall movement, as observed in previous studies (12).

The effect of elevated airway liquid on chest wall expansion before lung aeration is evident in Figure 4, where the relationship between chest wall expansion and external pressure is displaced towards a higher dimension. As a result, at the beginning of lung aeration, the chest wall may have been closer to its physiological expansion limit in *EL* kittens. Nevertheless, negative pressures increased chest wall expansion further which likely explains the much lower *C*_RS_ in *EL* compared to *Control* kittens (Figure 5). The increase in chest wall expansion in kittens exposed to external negative pressures likely includes flattening of the diaphragm, a feature that has previously been observed in *EL* kittens (3). Diaphragm flattening was significant in *EL* kittens, but not in *Control* kittens, indicating that the need to accommodate larger volumes of liquid within lung tissue is a major contributor to chest wall expansion. However, it is also possible that applying an external negative pressure to the abdomen reduced intra-abdominal pressures, allowing the apex of the diaphragm to be displaced into the abdomen, causing it to appear flattened.

It is also important to note that while external negative pressures were set to −6 cmH_2_O before lung aeration, the external pressures had increased to −2.7 ± 0.8 cmH_2_O after lung aeration. The increase in the external pressure applied was a result of chest wall expansion during lung aeration, which displaced liquid out of the main chamber of the plethysmograph and into the riser column, thus reducing the negative pressure. This change in external pressure associated with lung aeration was greatest in the external negative pressure group as the degree of chest wall expansion was greatest in these kittens. As we could not adjust the external pressure without disrupting the phase contrast X-imaging, kittens in the external negative pressure group were exposed to a lesser negative pressure than intended following lung aeration. Nevertheless, these reduced negative pressures had a marked effect on FRC levels, *C*_RS_, CO_2_ clearance, and the distribution of tidal ventilation.

### Respiratory function

Respiratory system compliance (*C*_RS_) is composed of both chest wall and lung compliance, but in the neonate, it is predominantly a measure of lung compliance (*C_L_*) as chest wall compliance (*C*_CW_) is so high $\left(\frac{1}{C_{\mathrm{RS}}}=\frac{1}{C_{L}}+\frac{1}{C_{\mathrm{CW}}}\right)$ (25). Thus, the increase and reduction in *C*_RS_ in *Control* kittens exposed to external negative and positive pressures, respectively, are likely due to changes in lung compliance. These changes could result from the effect that external negative and positive pressures had on FRC levels, placing the lung in more or less compliant regions of the pressure/volume curve (26).

As external negative pressures increased FRC levels in *EL* kittens, which would be expected to increase alveolar recruitment and gas exchange surface area, it is not surprising that expired CO_2_ levels were also increased in *EL* kittens. While expired CO_2_ levels were similar in *Control* and *EL* kittens exposed to external negative pressures, indicating greatly improved CO_2_ exchange in *EL* kittens, FRC levels were higher in *Control* kittens. These findings indicate that FRC levels in both groups had increased above the level at which CO_2_ exchange is surface area-limited, and are consistent with previous studies demonstrating the benefits of external negative pressures on oxygenation (12, 13, 23).

In contrast, external positive pressures decreased the expired CO_2_ levels in *Control* kittens in addition to reducing lung compliance. As CO_2_ is ∼27 times more soluble than oxygen, a reduction in the exchange of CO_2_ across the lungs indicates that oxygen exchange must also have been reduced as previously observed in lambs (12). Impaired oxygen exchange is consistent with the finding that external positive pressures reduce FRC levels in *Control* kittens, which is indicative of a reduced alveolar surface area available for gas exchange.

Interestingly, in *EL* kittens, external positive pressures did not reduce *C*_RS_, FRC, and expired CO_2_ levels compared to external atmospheric pressures, demonstrating that external positive pressures do not further impair respiratory function in kittens whose respiratory function is already compromised. Indeed, in kittens exposed to external positive pressure, *C*_RS_, FRC, and expired CO_2_ levels were similar in *EL* and *Control* kittens, demonstrating that these factors are negatively influenced by positive pressures, but that the impacts of *EL* and external positive pressures are not additive.

### External negative pressures vs. positive end-expiratory pressures

It is well established that PEEP plays an important role in both the creation and maintenance of FRC levels in mechanically ventilated preterm newborns at birth (27, 28). Without PEEP, the airways are prone to collapse and the distal airways reflood at end-expiration, which limits gas exchange to the period when the lungs are inflated as there is little to no air in the airway at FRC for gas exchange to occur (29). As external negative pressures are commonly thought to replicate PEEP (30, 31), we compared the effect of external negative pressures to different PEEP levels on FRC following lung aeration. We found that external negative pressures of approximately −3 cmH_2_O were able to replicate the effect of a PEEP of 4–6 cmH_2_O on FRC levels in both *Control* and *EL* kittens.

### Summary

We have shown that external pressures have a large impact on lung aeration, respiratory system compliance, and the distribution of ventilation within the lung in newborn near-term rabbit kittens. External positive pressures, which are not too dissimilar to the pressures applied to the chest by tight swaddling [5–10 cmH_2_O (11)] reduced *C*_RS_, FRC levels, expired CO_2_ levels, and redirected tidal ventilation into the upper lung lobes. In contrast, the application of external negative pressures (approximately −3 cmH_2_O) expanded the chest wall, and increased *C*_RS_, FRC levels, expired CO_2_ levels, and redirected tidal ventilation to the larger lower lung lobes. Additionally, we have shown that external negative pressures are as effective as a PEEP of 4–6 cmH_2_O in maintaining FRC levels. Thus, the results from this study indicate that external negative pressures may be an alternative approach for assisting near-term and term infants with respiratory distress.

### Limitations

We aimed to apply external negative pressures of −6 cmH_2_O in this study. However, aeration of the lungs and subsequent expansion of the chest wall displaced the water in the plethysmograph, thereby decreasing the magnitude of the external negative pressure applied. The chest wall expansion was greatest in the kittens exposed to external negative pressures, resulting in applied external negative pressures of approximately −3 cmH_2_O instead of the targeted −6 cmH_2_O. In contrast, the chest wall expanded less in kittens exposed to external atmospheric and positive pressures, so the applied pressures were closer to the intended levels of 0 and 6 cmH_2_O, respectively.

## Data Availability

The original contributions presented in the study are included in the article/Supplementary Material, further inquiries can be directed to the corresponding author.

## Supplementary material

Supplementary Video S1 Representative videos of lung aeration from the first breath after the sustained inflation ceased.

## References

[1] Reuter S Moser C Baack M. Respiratory distress in the newborn. Pediatr Rev. (2014) 35(10):417–28; quiz 29. 10.1542/pir.35-10-417 25274969 PMC4533247

[2] Chow SSW Creighton P Chambers GM Lui K. Report of the Australian and New Zealand Neonatal Network 2020. Sydney: UNSW Sydney, Australian and New Zealand Neonatal Network (2022).

[3] McGillick EV Lee K Yamaoka S Te Pas AB Crossley KJ Wallace MJ Elevated airway liquid volumes at birth: a potential cause of transient tachypnea of the newborn. J Appl Physiol. (2017) 123(5):1204–13. 10.1152/japplphysiol.00464.2017 28775070

[4] McGillick EV Pas ABT Croughan MK Crossley KJ Wallace MJ Lee K Increased end-expiratory pressures improve lung function in near-term newborn rabbits with elevated airway liquid volume at birth. J Appl Physiol. (2021) 131(3):997–1008. 10.1152/japplphysiol.00918.2020 34351817

[5] Yamaoka S Crossley KJ McDougall ARA Rodgers K Zahra VA Moxham A Increased airway liquid volumes at birth impair cardiorespiratory function in preterm and near-term lambs. J Appl Physiol. (2022) 132(4):1080–90. 10.1152/japplphysiol.00640.2021 35271407

[6] Davies IM Crossley KJ McGillick EV Nitsos I Rodgers K Thiel A Adverse respiratory patterns in near-term spontaneously breathing newborn lambs with elevated airway liquid volumes at birth. Front Pediatr. (2024) 12:1336154. 10.3389/fped.2024.1336154 38690521 PMC11058214

[7] Hooper SB Kitchen MJ Wallace MJ Yagi N Uesugi K Morgan MJ Imaging lung aeration and lung liquid clearance at birth. FASEB J. (2007) 12:3329–37. 10.1096/fj.07-8208com 17536040

[8] Siew ML Wallace MJ Allison BJ Kitchen MJ Te Pas AB Islam MS The role of lung inflation and sodium transport in airway liquid clearance during lung aeration in newborn rabbits. Pediatr Res. (2013) 73(1–4):443–9. 10.1038/pr.2012.197 23269118

[9] Hooper SB Fouras A Siew ML Wallace MJ Kitchen MJ te Pas AB Expired Co_2_ levels indicate degree of lung aeration at birth. PLoS One. (2013) 8(8):e70895. 10.1371/journal.pone.0070895 23951032 PMC3741323

[10] Siew ML Wallace MJ Kitchen MJ Lewis RA Fouras A Te Pas AB Inspiration regulates the rate and temporal pattern of lung liquid clearance and lung aeration at birth. J Appl Physiol (1985). (2009) 106(6):1888–95. 10.1152/japplphysiol.91526.2008 19342434

[11] Gerard CM Harris KA Thach BT. Physiologic studies on swaddling: an ancient child care practice, which may promote the supine position for infant sleep. J Pediatr. (2002) 141(3):398–404. 10.1067/mpd.2002.127508 12219062

[12] Diedericks C Crossley KJ Davies IM Riddington PJ Cannata ER Martinez OL Influence of the chest wall on respiratory function at birth in near-term lambs. J Appl Physiol. (2024) 136(3):630–42. 10.1152/japplphysiol.00496.2023 38328823 PMC11286272

[13] Grasso F Engelberts D Helm E Frndova H Jarvis S Talakoub O Negative-pressure ventilation. Am J Respir Crit Care Med. (2008) 177(4):412–8. 10.1164/rccm.200707-1004OC 18079496

[14] Cvetnic WG Cunningham MD Sills JH Gluck L. Reintroduction of continuous negative pressure ventilation in neonates: two-year experience. Pediatr Pulmonol. (1990) 8(4):245–53. 10.1002/ppul.1950080407 2196512

[15] National Health and Medical Research Council. Australian Code for the Care and Use of Animals for Scientific Purposes. 8th ed. Canberra: National Health and Medical Research Council (2013).

[16] Percie du Sert N Hurst V Ahluwalia A Alam S Avey MT Baker M The arrive guidelines 2.0: updated guidelines for reporting animal research. BMC Vet Res. (2020) 16(1):242. 10.1186/s12917-020-02451-y 32660541 PMC7359286

[17] Hooper SB Harding R. Fetal lung liquid: a Major determinant of the growth and functional development of the fetal lung. Clin Exp Pharmacol Physiol. (1995) 22(4):235–41. 10.1111/j.1440-1681.1995.tb01988.x 7671435

[18] Kitchen MJ Habib A Fouras A Dubsky S Lewis RA Wallace MJ A new design for high stability pressure-controlled ventilation for small animal lung imaging. J Instrum. (2010) 5(02):T02002. 10.1088/1748-0221/5/02/T02002

[19] Kitchen MJ Lewis RA Morgan MJ Wallace MJ Siew ML Siu KKW Dynamic measures of regional lung air volume using phase contrast x-ray imaging. Phys Med Biol. (2008) 53(21):6065–77. 10.1088/0031-9155/53/21/012 18843172

[20] Hadley L Flemmer AW Kitchen MJ Croughan MK Crossley KJ Lee KL Sustained inflation improves initial lung aeration in newborn rabbits with a diaphragmatic hernia. Pediatr Res. (2024) 95(3):660–7. 10.1038/s41390-023-02874-x 37952056

[21] Schneider CA Rasband WS Eliceiri KW. Nih image to imagej: 25 years of image analysis. Nat Methods. (2012) 9(7):671–5. 10.1038/nmeth.2089 22930834 PMC5554542

[22] Kitchen MJ Siew ML Wallace MJ Fouras A Lewis RA Yagi N Changes in positive end-expiratory pressure alter the distribution of ventilation within the lung immediately after birth in newborn rabbits. PLoS One. (2014) 9(4):e93391. 10.1371/journal.pone.0093391 24690890 PMC3972143

[23] Scharffenberg M Wittenstein J Herzog M Tauer S Vivona L Theilen R Continuous external negative pressure improves oxygenation and respiratory mechanics in experimental lung injury in pigs—a pilot proof-of-concept trial. Intensive Care Med Exp. (2020) 8(Suppl 1):49. 10.1186/s40635-020-00315-1 33336263 PMC7746426

[24] Papastamelos C Panitch HB England SE Allen JL. Developmental changes in chest wall compliance in infancy and early childhood. J Appl Physiol (1985). (1995) 78(1):179–84. 10.1152/jappl.1995.78.1.179 7713809

[25] Dreizzen E Migdal M Praud JP Magny JF Dehan M Chambille B Passive compliance of total respiratory system in preterm newborn infants with respiratory distress syndrome. J Pediatr. (1988) 112(5):778–81. 10.1016/s0022-3476(88)80702-9 3361391

[26] Henderson B Clotworthy N. Chapter 7—the management of volume loss. In: Harden B Cross J Broad M-A Quint M Ritson P Thomas S, editors. Respiratory Physiotherapy. 2nd ed. Edinburgh: Churchill Livingstone (2009). p. 85–100.

[27] te Pas AB Siew M Wallace MJ Kitchen MJ Fouras A Lewis RA Establishing functional residual capacity at birth: the effect of sustained inflation and positive end-expiratory pressure in a preterm rabbit model. Pediatr Res. (2009) 65(5):537–41. 10.1203/PDR.0b013e31819da21b 19190537

[28] Siew ML te Pas AB Wallace MJ Kitchen MJ Lewis RA Fouras A Positive end-expiratory pressure enhances development of a functional residual capacity in preterm rabbits ventilated from birth. J Appl Physiol. (2009) 106(5):1487–93. 10.1152/japplphysiol.91591.2008 19325025

[29] Hooper SB Siew ML Kitchen MJ te Pas AB. Establishing functional residual capacity in the non-breathing infant. Semin Fetal Neonatal Med. (2013) 18(6):336–43. 10.1016/j.siny.2013.08.011 24035400

[30] Easa D Mundie TG Finn KC Hashiro G Balaraman V. Continuous negative extrathoracic pressure versus positive end-expiratory pressure in piglets after saline lung lavage. Pediatr Pulmonol. (1994) 17(3):161–8. 10.1002/ppul.1950170305 8196996

[31] Borelli M Benini A Denkewitz T Acciaro C Foti G Pesenti A. Effects of continuous negative extrathoracic pressure versus positive end-expiratory pressure in acute lung injury patients. Crit Care Med. (1998) 26(6):1025–31. 10.1097/00003246-199806000-00021 9635650

